# Unreliable pulse oximetry in dark-skin patients: a plea for algorithm disclosure

**DOI:** 10.1186/s13613-022-00993-y

**Published:** 2022-02-22

**Authors:** Martin J. Tobin, Amal Jubran

**Affiliations:** Division of Pulmonary and Critical Care Medicine, Hines Veterans Affairs Hospital and Loyola University of Chicago Stritch School of Medicine, Hines, IL 60141 USA

We thank Drs. Ferrari and Scholkmann for their interest in our article [1].

The authors’ experience with the accuracy of noninvasive sensors in the face of elevated levels of carboxyhemoglobin and methemoglobin has considerable ramifications for the operation of pulse oximeters. If manufacturers have been able to make modifications—to hardware or software—to enable devices to overcome light-absorbance transmutations consequent to abnormal blood chemistry [2], there is every reason to expect that human ingenuity should be able to solve inaccurate pulse oximetry readings in patients with dark-skin pigmentation [3].

On a purely numerical basis, the problem of unreliable pulse oximetry readings in dark-skin patients is enormously greater than problems arising from abnormal levels of carboxyhemoglobin and methemoglobin. Given that hypoxemia is the lynchpin around which clinical decisions in seriously ill patients with COVID-19 revolve [4], it is imperative that caregivers are alert to the unreliability of pulse oximetry in dark-skin patients [3]. Clinical decisions based on faulty pulse oximetry readings have likely contributed to the several-fold greater number of deaths from COVID-19 in ethnic-minority patients than in white patients [5].

We do not know why manufacturers have invested considerable resources into solving the problems of oximetry in patients with abnormal blood chemistry, but have not made comparable endeavors into decrypting the riddle of unreliable pulse oximetry readings in patients of color. The contrasting experiences raise some unsettling questions.

We agree with Drs. Ferrari and Scholkmann that pulse oximetry manufacturers should make public the algorithms employed in their software. With these data, an imaginative investigator—perhaps far removed from the industry—may hopefully hit on a solution to the conundrum of deceptive pulse oximetry readings in patients with dark-skin pigmentation.

## Data Availability

Not applicable.
